# Naturopathic community clinics: an international cross-sectional survey

**DOI:** 10.1186/s12913-021-06806-5

**Published:** 2021-08-14

**Authors:** Iva Lloyd, Sophia Gerontakos, Valentina Cardozo

**Affiliations:** 1 World Naturopathic Federation, 20 Holly Street, Toronto, Canada; 2 NCNM, Southern Cross University, Military Rd, Lismore, NSW Australia; 3 Canadian College of Naturopathic Medicine, 1255 Sheppard Ave East, Toronto, Canada

**Keywords:** Naturopathy, Community clinic, Survey, Complementary therapies, Health promotion, Health services accessibility

## Abstract

**Background:**

Globally, naturopathic practitioners offer services in 98 countries, reaching every world region and providing care to diverse populations for a range of acute and chronic health conditions. Community clinics provide free or low-cost healthcare services and play a key role in providing necessary primary healthcare for underprivileged or marginalized populations. However, the reach and impact of naturopathic community clinics (NCCs) has not yet been examined. The aim of this study was to identify the characteristics of NCCs around the world, determine the types of services they offer and provide insight into the populations being served.

**Methods:**

Two online cross-sectional surveys were administered using purposive and snowball sampling. A 6-item screening survey was administered first to identify clinics and institutions who fit the criteria for NCC services, followed by a 40-item follow-up survey. Descriptive analysis was conducted using frequencies and means.

**Results:**

The screening survey returned a total of 37 responses from six world regions. Of those respondents who indicated involvement in NCCs, 74% went on to complete the follow-up survey. The majority of the responding NCCs were located in North America (50%), followed by Western Pacific (17%), Europe (10%), Asia (13%), Latin America (7%) and Africa (3%). The vast majority (71%) of the NCCs that have been in operation for more than 10 years are located in North America, while 43% of the NCCs that have been operational less than 5 years are in the Western Pacific Region. 80% of the responding NCCs were affiliated with a naturopathic school. The majority of respondents (76%) mentioned that they aim to serve underserved and/or marginalized populations, with 34% indicating that their target population is low-income families, 21% focusing on serving immigrants and refugees, 21% on serving people experiencing homelessness, 14% on serving Indigenous peoples, 14% on serving those with specific gender differences, 10% on serving seniors and 10% on serving drug users.

**Conclusion:**

The naturopathic profession offers free or significantly low-cost naturopathic services through community clinics around the world. The findings of this survey provide insight into the important role of the naturopathic profession in primary health care and provide rationale for exploring this topic in greater detail.

## Background

Naturopathy is a traditional system of health care based on core philosophical principles that uses an eclectic range of natural therapies delivered by medically trained practitioners [1]. Currently, naturopathic practitioners (NPs) offer naturopathic services in 98 countries [2], reaching every world region [3]. Globally, NPs provide care to diverse populations for a range of acute and chronic health conditions [4]. Health promotion and disease prevention – key aspects of primary health care [5] – are core principles of the naturopathic profession with NPs emphasizing lifestyle-based self-care, nutrition, preventive behaviors, physical activity and stress management counselling alongside therapeutic medicaments (such as herbal medicine, clinical nutrition, homeopathy), and manual therapies [4].

The naturopathic profession is actively engaged in community health education, however, the extent to which this is occurring through community clinics is not known. Ensuring healthcare to marginalized, low-income and underserved populations is an important aspect of primary health care and is often achieved through health promotion and disease prevention strategies offered by community clinics. Community clinics (also known as community health centers) provide free or low-cost healthcare services and play a key role in providing necessary healthcare for the underprivileged, low income and specialized groups offering healthcare that is accessible, culturally competent and patient centered [6]. Naturopathic community clinics (NCCs) are community clinics that offer naturopathic services. To date, there is a gap in research exploring the role of naturopathy in community health centers, however there is some evidence that naturopathic services are being delivered in these settings [7].

Naturopathic educational institutions in various countries around the world provide services at NCCs which double as teaching clinics [8–10]. However, the reach of those NCCs and the extent to which NPs are involved in providing services to low-income, marginalized and underserved populations has not been studied. Thus, the aim of this study was to identify the characteristics of NCCs around the world, determine the types of services they offer and provide insight into the populations being served. A more in-depth understanding of the reach of NCCs in community healthcare will provide a foundation for future research to examine the role of NCCs which may be an under-recognized or under-utilized health care resource. As such, this research provides the first international examination of naturopathic services offered through NCC’s and explores variations by world region.

## Methods

### Study design and content

A cross-sectional, descriptive study design was employed to achieve the research aim. This study is a secondary analysis of two online cross-sectional surveys conducted by the World Naturopathic Federation (WNF). The data was organizational in nature and was originally gathered as an informal activity by the WNF (not for research purposes), after which, the research team were given permission by the WNF to access and analyse it. The inclusion criteria for this study (as ascertained by the screening survey) was clinics that offer naturopathic services to patients free of charge or at a substantially discounted price anywhere in the world. Clinics who did not fulfil these criteria were excluded. Survey design was informed by previous surveys conducted by the WNF, as well as by an international survey of NP practice behaviors [4, 11, 12]. The surveys were piloted for content and layout with two educational members and two full members from the WNF and modifications were made accordingly. The surveys were distributed in English language only.

The screening survey identified which naturopathic institutions fit the criteria of NCC. It contained 6-items covering basic demographic information about the clinic and involvement with community clinic(s) offering naturopathic services and took approximately 3 minutes to complete. The community clinic services follow-up survey consisted of 40-items covering nine domains: demographic information, basic information about the NCC (including its affiliation with a naturopathic school and the length of time it has been in operation), patient demographics, funding, consultation models, marketing, conditions and treatments offered, inter-professional collaboration and basic information about the individual filling the survey. This survey took approximately 25 min to complete. Both surveys were completed by staff clinicians or clinic administrative personnel (not by patients). Consent to participate in both surveys was implied by survey completion. Implied consent is a typical consent-type for survey studies [13, 14].

### Recruitment and administration

Participants for the screening survey were recruited using purposive and snowball sampling via WNF listing of the naturopathic educational institutions internationally, and full member organizations representing naturopathic professional associations in 35 countries. The WNF distributed an email to 85 naturopathic educational institutions with a link to the survey and a separate email to full members asking them to share the request to participate with their members. Access to the survey was open to any individual accessing the link in the invitation to participate. The screening survey was also shared on multiple social media platforms via both professional naturopathic organizations and private naturopathic stakeholders. Data for the screening survey was collected between the 20th of September 2019 and the 14th of January 2020. Respondents who indicated that their institution participated in NCCs were then sent a link to the community clinic services survey via email. Data for the follow-up survey was collected from November 28th, 2019 until February 18th, 2020. A second request for participation was sent on July 18th with responses received until July 30th, 2019. Participation in both surveys was voluntary and non-incentivized.

### Data cleaning and analysis

The 40 responses from the screening survey were downloaded as a Microsoft Excel file from Survey Monkey. Duplicate responses were removed leaving a total of 37 responses. Other than contact name, the information from the duplicate responses was identical. Four of the respondents from Australia indicated their world region to be Asia. According to the WNF and the World Health Organization (WHO), Australia belongs to the world region of the Western Pacific. The responses were changed to reflect this. Based on demographic information being collected, it was possible to categorize the NCCs as being either educational institutions, full member organizations or practitioner clinics. Similarly, 31 responses from the community clinic services follow-up survey were downloaded as a Microsoft Excel file from Survey Monkey. One duplicate was removed leaving a total of 30 responses. Descriptive statistics including frequencies and means were used to explore the data.

## Results

### Screening survey

The response rate from the naturopathic educational institutions was 28%. Of those that started the screening survey 100% finished all 6-items. A total of 37 responses were received from six world regions with 24% from each Europe and North America, 22% from each Latin America and Western Pacific, 5% from Asia and 3% from Africa (Table 1). The vast majority of the respondents (65%) were from naturopathic educational institutions, followed by those from individual naturopathic clinics (24%), with the remaining 8% belonging to national organizations. All responses from national naturopathic organizations were from Latin America. All of the responses from Asia (*n* = 2) and Africa (*n* = 1) corresponded to naturopathic educational institutions. The vast majority (75%) of practitioner clinic responses were from Western Pacific.

**Table 1:** Screening Survey: respondents by world region and institution type

Category	All n (%)	Offering NCCs n (%)	Naturopathic Schools n (%)	Practitioner Clinics n (%)	National Organizations n (%)
Africa	1 (3)	1 (100)	1 (100)	0 (0)	0 (0)
Asia	2 (5)	2 (100)	2 (100)	0 (0)	0 (0)
Europe	9 (24)	5 (56)	8 (89)	1 (11)	0 (0)
Latin America	8 (22)	4 (50)	5 (63)	0 (0)	3 (38)
North America	9 (24)	8 (89)	7 (78)	2 (22)	0 (0)
Western Pacific	8 (22)	7 (88)	2 (25)	6 (75)	0 (0)
Total	**37 (100)**	**27 (73)**	**24 (65)**	**9 (24)**	**3 (8)**

Of the 37 respondents, 73% (*n* = 27) indicated that they participated in one or more community clinics offering naturopathic services, with a total of 96 NCCs being represented by respondents (Table 2). The majority of responses from NCCs were from North America (30%), followed by Western Pacific (26%), Europe (19%), Latin America (15%), Asia (7%) and Africa (3%). 100% of the respondents from Africa and Asia indicated that they offered NCCs. Furthermore, there was an average of 3.6 NCCs per respondent. North American had the highest average with 6.1 NCCs per respondent followed by Asia (4.5) and Latin America (3.3). On the other hand, Europe and Western Pacific had the lowest number of NCCs per respondent with 1.8 and 1.9 NCCs per respondent accordingly.

**Table 2:** Screening Survey: respondents offering NCCs by world region

World Region	Distribution of NCC respondents n (%)	Total NCCs represented n (%)	Average number of NCCs per respondent
Africa	1 (4)	3 (3)	3.0
Asia	2 (7)	9 (9)	4.5
Europe	5 (19)	9 (9)	1.8
Latin America	4 (15)	13 (14)	3.3
North America	8 (30)	49 (51)	6.1
Western Pacific	7 (26)	13 (14)	1.9
Total	**27 (100)**	**96 (100)**	**3.6**

### Naturopathic community clinic services follow-up survey

Out of the 27 respondents that indicated their involvement in NCCs, 20 (74%) went on to complete the community clinic services survey. In addition, four institutions submitted multiple entries (for their various community clinics) accounting for a total of 30 responses representing 71 NCCs. Of the 30 respondents 27% indicated that they had been affiliated with the community clinic more than 10 years, 43% 6–10 years, 10% 2–5 years and 20% less than 2 years.

#### NCC demographics

Details of the demographic characteristics of respondents offering NCCs are shown in Table 3. The majority of the responding NCCs were located in North America (50%), followed by Western Pacific (17%), Asia (13%), Europe (10%), Latin America (7%) and Africa (3%). Over half (57%) of the respondents indicated that the NCC has been operational for more than 10 years, with the remainder evenly split between NCCs that have been operational for 6–10 years (20%) and less than 5 years (23%). The vast majority (71%) of the NCCs that have been in operation for more than 10 years are located in North America, while 43% of the NCCs that have been operational less than 5 years are in the Western Pacific Region. 83% of the total responding NCCs indicated that they are affiliated with a naturopathic school. When asked to select a category to describe the community clinic, the majority of the respondents (80%) indicated that the NCC is a teaching clinic associated with a naturopathic school. The remaining respondents identified as NCCs offered by a naturopathic practitioner (13%) or relief work associated with a non-profit organization (7%).

**Table 3:** Demographic characteristics of respondent offering NCCs

Characteristic	Category	n	%
World Region	Africa	1	3
	Asia	4	13
	Europe	3	10
	Latin America	2	7
	North America	15	50
	Western Pacific	5	17
Time period that naturopathic services have been offered for	Less than 5 years	7	23
	6–10 years	6	20
	More than 10 years	17	57
Affiliation with naturopathic school	Yes	25	83
	No	5	17
NCC category	Teaching clinic associated with naturopathic school	24	80
	Community Clinic offered by naturopathic practitioner	4	13
	Relief work associated with a non-profit organization	2	7
NCC location	Within the community of target population	20	67
	Within a school	4	13
	Stand alone	4	13
	Within a private naturopathic clinic	2	7

#### NCC patient demographics

When asked an open-ended question about the NCC’s target population, the majority of respondents (76%) mentioned that the NCC aims to serve underserved and/or marginalized populations; with 34% indicating that their target population is low income families, 21% focusing on serving immigrants and refugees, 21% on serving people experiencing homelessness, 14% on serving Indigenous peoples, 14% on serving the lesbian, gay, bisexual, transgender, queer and/or questioning, two-spirit, intersex and asexual (LGBTQ2SIA+) community, 10% on serving seniors and 10% on serving drug users, 7% on serving people living with HIV and AIDS, 7% on serving people with mental health disorder, 7% on serving those seeking relief from domestic violence, 3% on individuals seeking palliative care, and 3% on serving teens. As shown in Tables 3, 67% of the respondents indicated that the NCC "often" is located within the corresponding community of the target population.

Table 4 outlines the age and gender distribution of patients served at NCCs, showing a wide range of variation between respondents. Females patients appear to be most commonly served, making up on average 63 ± 20.5% of the patient base, ranging from 19 to 98%. Non-binary and transgender patients were reported to make up on average 10 ± 7.7% and 7 ± 6.5% of the patient base respectively. The age groups of 40–49 and 50–59 represent the largest proportion of the patient base, accounting for 23 ± 14.4% and 23 ± 11.7% respectively; followed by 30–39 year old’s at 17 ± 10.9%, 19–29 year old’s at 15 ± 14.2% and 65+ year old’s at 15 ± 16.2%. Children under 19 years old represented under 9% of the patients seen in the NCC with an average of 4 ± 4.3% age 13–18 years and 5 ± 5.8% age 0–12 years.

**Table 4:** Gender and age distribution of NCC patients

Category	Average ± SD (%)	Median (%)	Range (%)	Total responses^a^
Gender				
Female	63 ± 20.5	65	19–98	29
Male	29 ± 14.8	28	2–70	29
Non-binary	10 ± 7.7	10	1–25	11
Transgender	7 ± 6.5	5	1–25	13
Other	3 ± 1.7	2.5	1–5	6
Age (years)				
0–12	5 ± 5.8	3	0–20	28
13–18	4 ± 4.3	2	0–15	28
19–29	15 ± 14.2	10	0–69	28
30–39	17 ± 10.9	15	0–50	28
40–49	23 ± 14.4	20	1–70	28
50–59	23 ± 11.7	20	0–50	28
65+	15 ± 16.2	10	0–60	28

^a^ Total responses per item. Total respondents n = 30

#### Clinic funding and operation

The most common funding source of NCCs was donations as indicated by 60% of respondents, followed by private funding (40%). Only 23% of the respondents indicated that they received government funding. In addition, 23% of participants indicated receiving funding from other sources including tuition and patient contributions.

Table 5 presents information about the provision and funding of consumable products that are used at NCCs. 87% indicated that they use botanical medicines, 83% nutraceuticals, 63% homeopathics and 63% flower essences. Comments in the other category (23%) included acupuncture needles, moxa, ear seeds, essential oils and mud therapy. 40% of the respondents indicated that patients do not pay for consumables, 37% indicated that patients pay a fee and 23% selected “other”, indicating that patients pay a reduced or minimal fee based on need or that availability of free consumables depends on donations. This is reflected in the responses to the open-ended question “Where do your consumables come from?” with the majority of respondents noting that consumables primarily come from donations in kind (58%), and monetary donations (35%).

**Table 5:** Provision and funding of consumable products at NCCs

Characteristic		n	%
Consumable products used/dispensed	Botanical medicines	26	87
	Nutraceuticals	25	83
	Homeopathics	19	63
	Flower essences	19	63
	Other	7	23
Do patients pay for consumables?	Yes	11	37
	No	12	40
	Other	7	23
Funding sources of consumables	Donations in kind	15	58
	$ value donations	9	35
	Patients pay	5	19
	Other	4	15

Total respondents *n* = 30.

#### Consultation models and staff

Table 6 shows the consultation, staffing and marketing characteristics of the NCCs revealing that 80% of the naturopathic consultations are done as one-on-one visits; with 87% of all appointments being scheduled ahead of time. When asked about the number of patients generally seen per day, 38% of respondents indicated seeing up to 9 patients, 31% seeing 10–19, and 10% seeing over 30 patients. Nonetheless, the number of patients per day varied between 1 and 250 (with 250 patients per day being representative of a NCC within a hospital in India). 76% of the NCC respondents indicated that their staff consists of four or fewer NPs and nine or fewer naturopathic students; with the number of students per qualified NP being 5–9 39% of the time and 1–4 36% of the time. 72% of respondents indicated that the compensation for the NPs is fully or partly covered by the affiliated school, 24% that the NPs donate all or some their time, and 21% that the community clinic compensates the NPs for some or all of their time.

**Table 6:** NCC Consultation, staffing and marketing characteristics

CONSULTATION		n	%
Consultation model	One-on-one	24	80
	Group consultation	0	0
	Both (one-on-one and group consultation)	6	20
Appointment management	Scheduled	26	87
	Referred to clinic by other health practitioners	14	47
	Walk-in	16	53
NCC Service frequency	Daily	8	27
	Weekly	16	53
	Monthly	3	10
	Periodically	3	10
Number of patients seen per day	0–9	11	38
	10–19	9	31
	20–29	6	21
	30+	3	10
	Minimum	1–2	N/A
	Maximum	200–250	N/A
STAFFING		**n**	**%**
NPs present at the NCC	1–4	22	76
	5–9	4	14
	10+	3	10
Naturopathic students present at the NCC	None	6	21
	1–9	18	62
	10–19	3	10
	20+	2	7
Number of students per qualified NP	N/A	6	21
	1–4	10	36
	5–9	11	39
	10+	1	4
Compensation modes for NPs/supervisors	Paid by affiliated naturopathic school	21	72
	Paid by the Community Clinic	6	21
	Donate their time	7	24
Requirements for NPs working at NCCs	Cultural competency	16	53
	Specialty-related training	8	27
	Staff at the affiliated naturopathic institution	18	60
	Licensed naturopath / naturopathic doctor	28	93
MARKETING		**n**	**%**
Marketing strategies used to attract patients	External marketing	14	47
	Internal marketing	18	60
	Street signage	7	23
	Flyer drops	5	17
	Internet/social media/webpage	18	60
	Mailing list	8	27
	Referral	11	37
	Word of mouth	28	93

Total respondents *n* = 30.

Cultural competency training was reported as prerequisite for working at the NCC by 53% of the respondents. 93% of respondents also indicated that NPs had to be licensed in order to work at the NCC. In a follow-up open ended question, respondents indicated that additional requirements may include: second language fluency, clinical experience, and/or a working with children check. When it comes to marketing strategies used to attract patients to the NCCs word of mouth is the most common at 93%, followed by the use of internal marketing and internet/social media/webpage, each at 60%.

Table 7 shows the duration of appointments at NCCs. The time for initial visits ranged from 15 to 90 min, with 75% of respondents indicating that the initial visits usually last 60 to 90 min. The time for follow-up visits ranged from 15 to 60 min, with 54% of respondents indicating that the follow-up visits usually last 45–59 min. The time for acute and walk-in visits ranged from 15 to 60 min, with 44% of respondents indicating that acute and walk-in visits usually last 45–59 min.

**Table 7:** NCC appointment duration

Appointment Type (Total Respondents)	Average (min)	Median (min)	Range (min)
Initial (n=26)	60	60	15–90
Follow-up (n=24)	41	45	15–60
Acute/Walk-in (n=16)	37	43	15–60

#### Conditions treated and treatments used

**Fig. 1:**
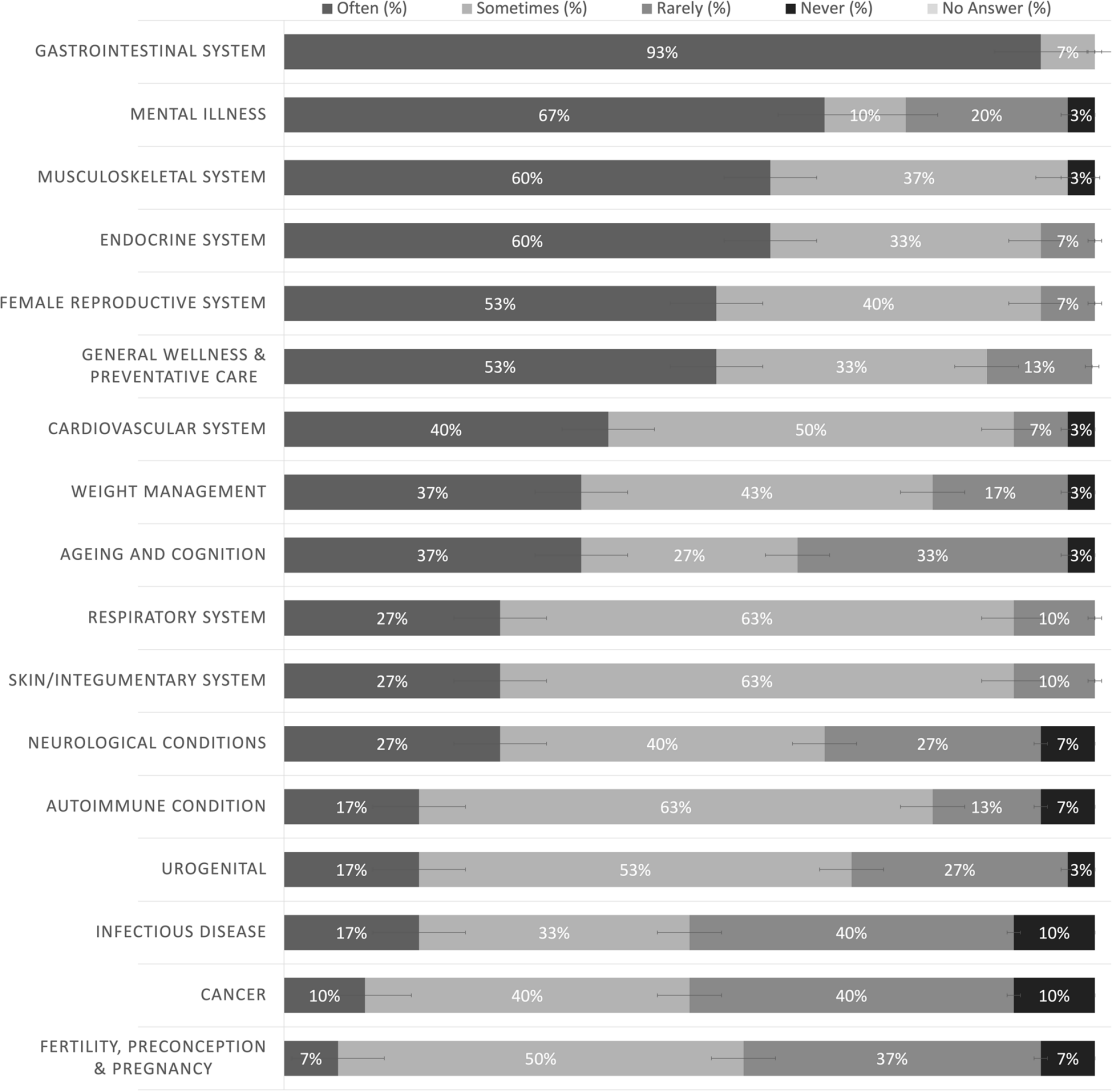
Frequency of Health Conditions for patients at NCCs

The respondents indicated that on average 56 ± 25% of the patients that visit the NCC for naturopathic care do so for chronic complaints or conditions, 27 ± 20% for acute care and 15 ± 10% for general health management. Figure 1 outlines the frequency that patients present with various health conditions as estimated by the respondents. When asked “how often do the patients visiting the community clinic present with the following complaint/concern”, gastrointestinal complaints were the most common with 93% of respondents selecting “often”. This was followed by mental illness concerns (with 67% of respondents selecting “often”) and endocrine and musculoskeletal complaints (with 60% of respondents selecting “often”). 77% of respondents indicated that patients presented with at least 10 out of the 17 complaint options “sometimes” or “often”. The complaint that was the least reported was infectious diseases with 50% of respondents selecting “often” or “sometimes”.

**Fig. 2:**
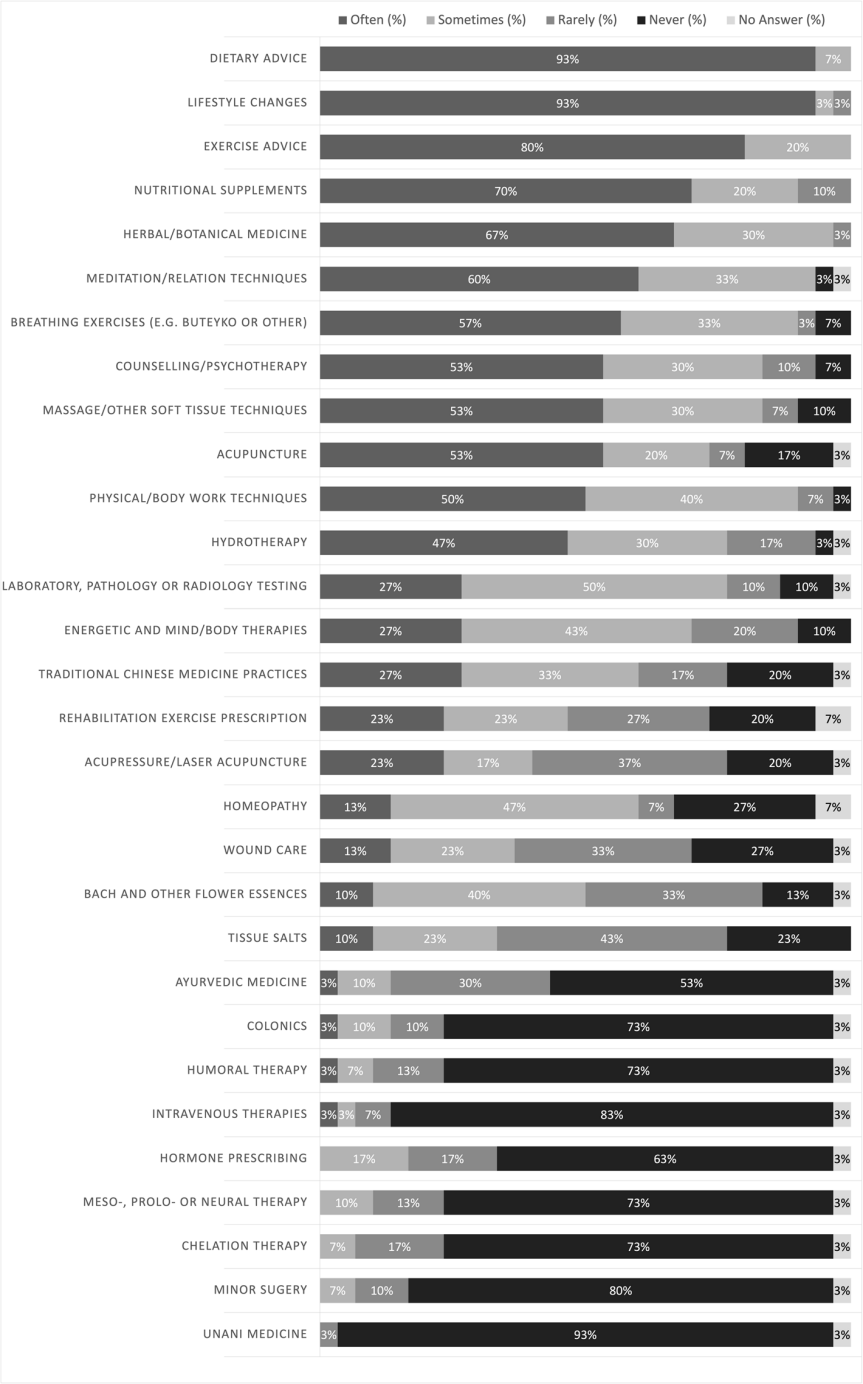
Frequency of treatments prescribed, suggested or recommended by NPs at NCCs

Figure 2 outlines the rate that treatments are performed, prescribed, suggested or recommended at the community clinic by the NPs (as estimated by the respondents). When asked “how often are the following treatments performed, prescribed, suggested or recommended within the community clinic by the naturopathic practitioners?” the most common treatments recommended were dietary and exercise advice, with 93 and 80% of respondents selecting “often” respectively. When looking at the responses for the 25 treatment options included in the survey, 80% of the respondents identified that 10 of the treatments were prescribed “often” or “sometimes”; another 6 treatments were reported as being prescribed “often” or “sometimes” by 60% or more of the respondents; and 8 of the treatment options were “never” prescribed as indicated by 63% or more of the respondents.

When asked if NPs offered other programs besides naturopathic consultations 50% of respondents indicated that they did. In a follow-up open ended question they indicated that additional programs offered include cooking classes, mindfulness and meditation classes, talks at local women’s shelters, aerobics and yoga, aquatic therapy, National Acupuncture Detoxification Association protocol for substance use, and educational workshops or groups about various health topics ranging from living with hepatitis C to transgender health, diabetes and herb-drug interactions.

#### Interprofessional collaboration

Figure 3 presents the estimated frequencies that patients of the NCC see other medical/health care practitioners for their primary presenting complaint. The majority of respondents (63%) indicated that patients "often" see a general practitioner, family physician or medical doctor for their primary presenting complaint; followed by other allied health practitioners including psychologist, physiotherapists, and dieticians (47%). Approximately 40% of respondents indicated that medical specialists as well as other complementary medicine practitioners including acupuncturist, massage therapists and homeopaths were "often" consulted for the primary presenting complaint. Only 10% of respondents indicated that the NCC "often" served as the primary health care provider for the presenting complaint.

**3:**
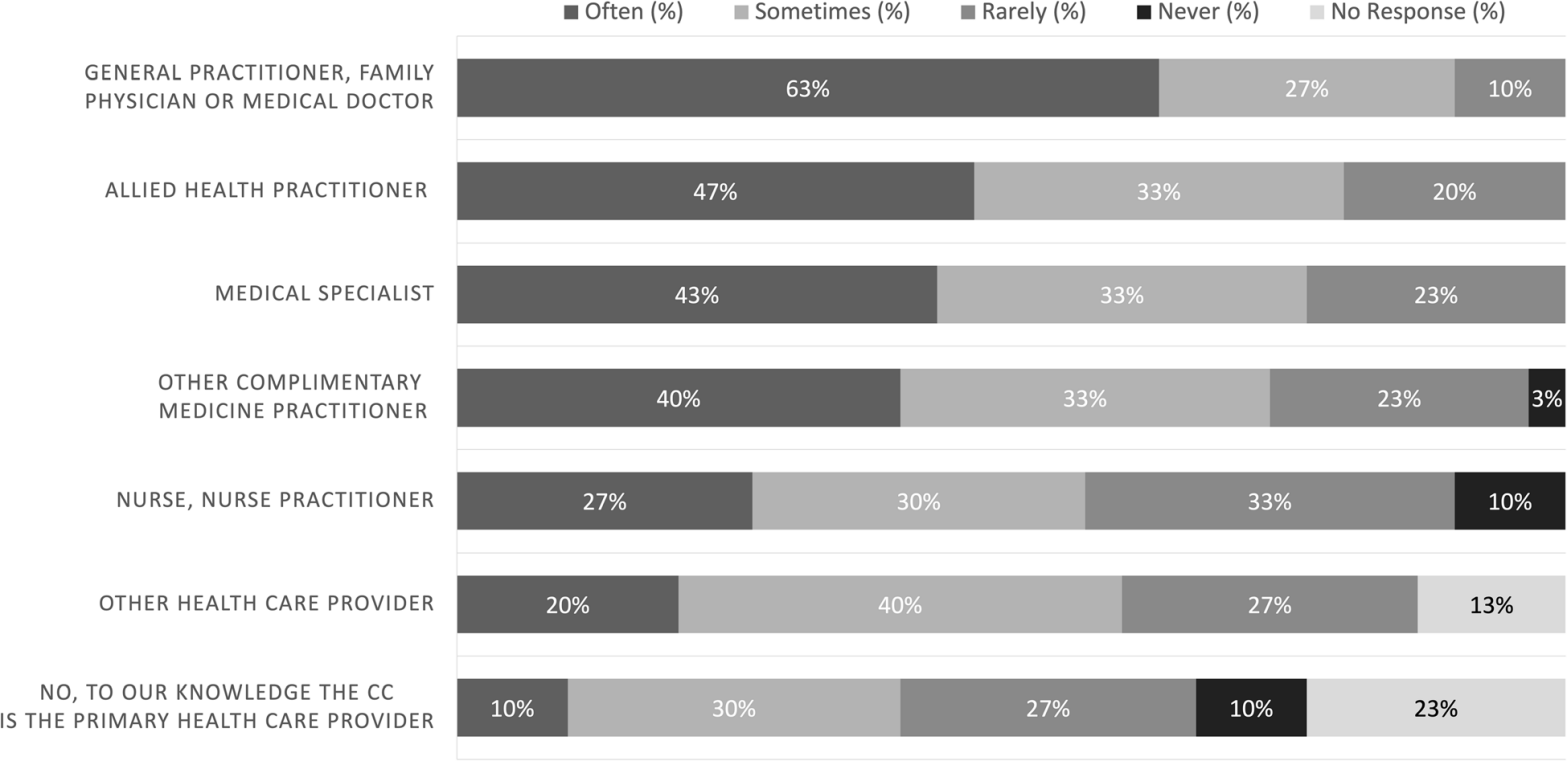
Frequency at which NCC patients see other health care practitioners for their primary presenting complaint

Table 8 shows the proportions of NCCs that offer services from health care practitioners other than NPs. Counsellors and nurse/nurse practitioners were the most common health care practitioners present at NCCs as indicated by 47 and 43% of respondents respectively. General medical practitioners were reported as being part of the NCC by 33% of the respondents.

**Table 8:** Additional health care practitioners that offer services within NCCs

Health care practitioner	n	%
Counsellor	14	47
Nurse, nurse practitioner	13	43
Dietician	12	40
Nutritionist	12	40
Psychologist or Psychotherapist	11	37
Massage therapist	11	37
General practitioner (GP), family physician or medical doctor	10	33
Acupuncturist	9	30
Physical therapist	8	27
Yoga practitioner	6	20
Other	5	17
Homeopath	2	7
Pilates practitioner	1	3

Total respondents *n *= 30.

## Discussion

This study is the first of its kind to examine the characteristics and reach of NCCs globally. NCCs have been a component and offering of various naturopathic educational institutions for over three decades [8, 9] and the results of this study suggest there is substantial growth of NCC offerings by not only educational institutions globally but also in the areas of relief work, private practices and practitioners. The results of this study suggest that the majority of schools offering NCCs are located in North America with naturopathic schools in Africa, Asia and Western Pacific providing NCCs more recently. In the Western Pacific (where few NCCs were reported as being associated with a school) there was higher prevalence of private practitioners offering NCCs. As the survey topic for this study was self-selecting, further research is needed to determine the commonality and source of NCCs in each world region and whether the prevalence of private NCCs in some world regions is potentially filling a gap where there is a lack of institution-based NCCs.

The study delivers a number of key findings providing insight into the functionality and practicality of NCCs as well as the demographics and characteristics of populations served and treatments offered. A number of these findings align with other research examining characteristics of naturopathic practice [4], complementing a growing body of evidence and providing insight into key areas requiring further research. In patient demographics, NCCs report serving more female patients which is consistent with previous research on international naturopathic practice [4]. In addition to the similarities in findings of gender, this study also presents a new finding – that the naturopathic profession (specifically NCCs) is serving trans-gender and non-binary persons – genders that have not been reported on in previous naturopathic literature. Furthermore, NCCs appear to serve patients across a broad range of ages (covering nearly all ages) and for a broad range of conditions with an emphasis on the gastrointestinal system, mental health, endocrine, musculoskeletal system and general health and wellbeing – aligning with other naturopathic practice research [4]. Additionally, the treatments being used in NCCs correspond with other research on international naturopathic practice with an emphasis on dietary advice, exercise advice, nutritional supplements and herbal and botanical medicine [4]. The results indicate the most common consultation model used in NCCs is a one-on-one model including a longer initial appointment (approximately 60 min) followed by shorter follow-up appointments (approximately 30–45 min), aligning with other reports highlighting the longer nature of naturopathic consultations emphasizing the patient-centered approach implemented by NPs [15]. However, 20% of NCCs also reported using group consultations (in addition to one-on-one) – a model not often reported in general naturopathic practice, but becoming more common with medical and other integrative medicine practitioners for reaching diverse and underserved communities and addressing healthcare disparities [16, 17]. Further research is needed to determine the reasons for and impact of using a group model within NCCs.

Although many of the characteristics of consultations and treatments are similar in NCCs to general naturopathic practice, the populations being served appear to differ. The results of this study show that NCCs are reaching underserved and/or marginalized populations including low-income families, immigrants and refugees, homeless people, Indigenous peoples, LGBTQ2SIA, senior citizens and drug users as well as a smaller number of people living with HIV and AIDS, palliative care patients and victims of domestic violence. Population-specific details haven’t been reported in other naturopathic practice research, however an Australian study reporting on patient demographics found over 40% of patients who consult with a naturopath have a university degree, over 60% work full-time and 65% do not have a healthcare card (a card provided to people on unemployment, disability or low-income benefits in Australia), suggesting patients who consult with naturopaths in private practice are more often from middle or upper socio-economic demographics [14]. In comparison, it appears NCCs may be reaching different populations who may not otherwise access the care-type in a private practice setting. Over 50% of the NCCs also reported cultural competency as a practitioner prerequisite for working in the clinic, suggesting population-specific services and a level of sensitivity to populations being served. Despite the demand for NCCs and the diversity in populations served, only 23% of NCCs report receiving government funding, with at least 60% of NCCs funded by donations. The lack of funding combined with demand and diversity in reach of NCCs shows more research is needed to explore suitable funding models.

The findings of this study should of course be interpreted and applied within the context of the study’s limitations. While this is the most comprehensive global study to date examining the characteristics of NCCs, it may not be generalizable to the entirety of NCCs across the international naturopathic profession; however, it provides preliminary data and a basis for more extensive and focused studies on global NCCs. The recruitment strategy had an emphasis on targeting global naturopathic educational institutions for a comprehensive analysis of institution-run NCCs and may bias the results towards participants associated with educational institutions and those who may be more academic whilst potentially excluding smaller organizations or individual naturopathic clinics and practitioners who offer NCC services. Additionally, the survey was only administered online and in the English language, which may have introduced bias towards clinics, institutes and organizations with access to a computer and English-language proficiency – other NCCs who don’t fulfil this criteria may have differing characteristics and demographics. In particular, a language barrier may have contributed to the relatively low response rate of the preliminary screening survey. By translating the survey tool into additional languages this limitation could be remedied in future research to collect more comprehensive data on global NCCs. Further, the survey items required participants to self-report and report on clinical characteristics which were not independently verified by the researchers and may have introduced additional bias. Despite these limitations, the number of locations represented (more than five) and the number of participating clinics (more than 15) in this study is sufficient to provide a level of representativeness [18], and the study is an important contribution to the understanding of the reach and impact of global NCCs.

## Conclusion

The naturopathic profession offers free or significantly low-cost primary naturopathic healthcare through community clinics around the world. The naturopathic services offered in these community clinics are representative of general naturopathic practice, but appear to be reaching different populations including marginalized, low-income and underserved populations. The contribution of naturopathic care to these underserved populations has generally been overlooked, but as the number and countries providing NCCs increases this will likely change. The majority of these NCCs are affiliated with naturopathic educational institutions, but there is a growing trend in some world regions for individual NPs in private practice to offer their services through NCCs. If the prevalence of individual naturopathic practitioner NCCs increases, this could impact regulatory standards in those countries where naturopathic regulation is in place. Assessing access of healthcare services to those individuals that are marginalized and underserved is an important aspect of primary health care and is an area of increasing focus in the global naturopathic community. The findings of this survey provide insight into the important role of the naturopathic profession in primary health care and provide rationale for exploring this topic in greater detail.

## Data Availability

The anonymized datasets analyzed during the current study are available from the corresponding author on reasonable request.

## Abbreviations

LGBTQ2SIA+: lesbian, gay, bisexual, transgender, queer and/or questioning, two-spirit, intersex and asexual
NCCs: Naturopathic community clinics
NPs: Naturopathic practitioners
WHO: World Health Organization
WNF: World Naturopathic Federation
